# Infected aryepiglottic cyst as a cause of persistent sore throat: case report

**DOI:** 10.1016/j.bjorl.2026.101876

**Published:** 2026-08-12

**Authors:** Gustavo Polacow Korn, Fernando Pochini Sobrinho, Hugo Luis de Vasconcelos Chambi Tames

**Affiliations:** a Hospital Albert Einstein, São Paulo, SP, Brazil; b Universidade Federal de São Paulo, São Paulo, SP, Brazil; c Universidade de São Paulo, São Paulo, SP, Brazil

Laryngeal cysts represent a diverse collection of pathologic entities that account for 5% of nonmalignant laryngeal lesions.^1^ Laryngeal cysts are generally more prevalent in men, although women appear to present with symptoms more frequently.^1^ Aryepiglottic cyst is not a common find in patients with laryngeal complains. Some case reports showed cases with respiratory distress due to the size of the cyst. One report described a case in which the cyst was valving and blocking the view of his vocal cords.^2^ One case described a young male who developed dyspnea hours after tracheal intubation.^3^ Another case report showed a case with an elderly patient which presented sore throat and foreign body sensation and symptoms improved after non-surgical treatment.^4^ And other publication reported a case with aryepiglottic fold cyst causing obstructive sleep apnea syndrome.^5^ We report a case of a young female with persistent sore throat despite treatment for acute tonsillitis. This case emphasizes that persistent sore throat warrants further investigation, even after the oropharyngeal examination improves.

## Case report

**Fig. 1:**
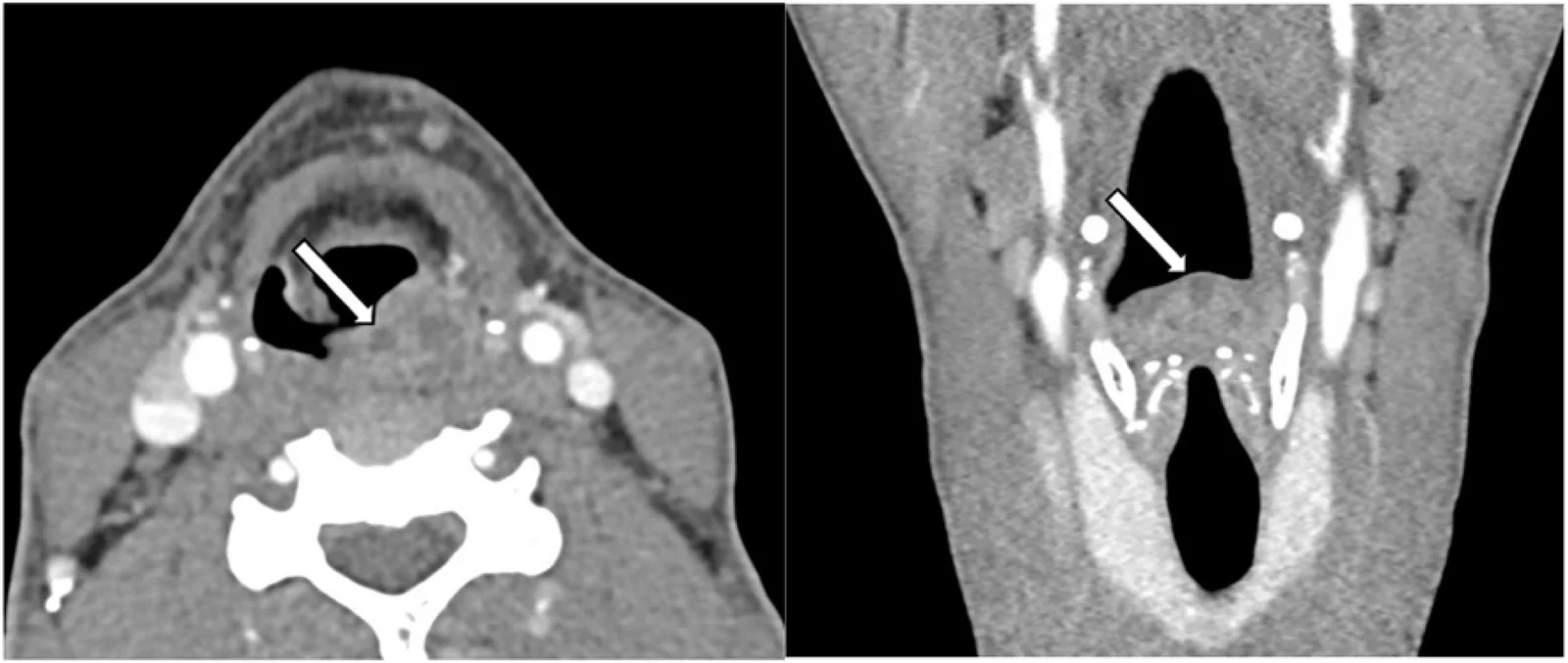
Axial and coronal post-contrast CT images before the first hospitalization demonstrate diffuse thickening and heterogeneous enhancement of the aryepiglottic folds, more pronounced on the left, where a poorly defined oval hypoattenuating area is observed (arrows).

A 33-year-old female software architect with a two-week history of sore throat presented to the emergency room with a diagnosis of acute bacterial tonsillitis and received benzathine penicillin. She had prior history of recurrent tonsillitis. With no improvement, she returned to the emergency room in the following days. Four days after the first antibiotic, the patient experienced severe sore throat, but in the central region of the neck, despite improvement in the appearance of the oropharynx. On that day, a neck CT scan was performed, revealing diffuse thickening of the aryepiglottic folds, more pronounced on the left side, with increased and heterogeneous post-contrast enhancement, compatible with inflammatory or infectious changes. Additionally, a poorly defined, oval-shaped hypoattenuating area without significant enhancement was noted along the medial aspect of the left aryepiglottic fold (Fig. 1). Amoxicillin associated with clavulanate, and oral corticosteroids were administered.

**Fig. 2:**
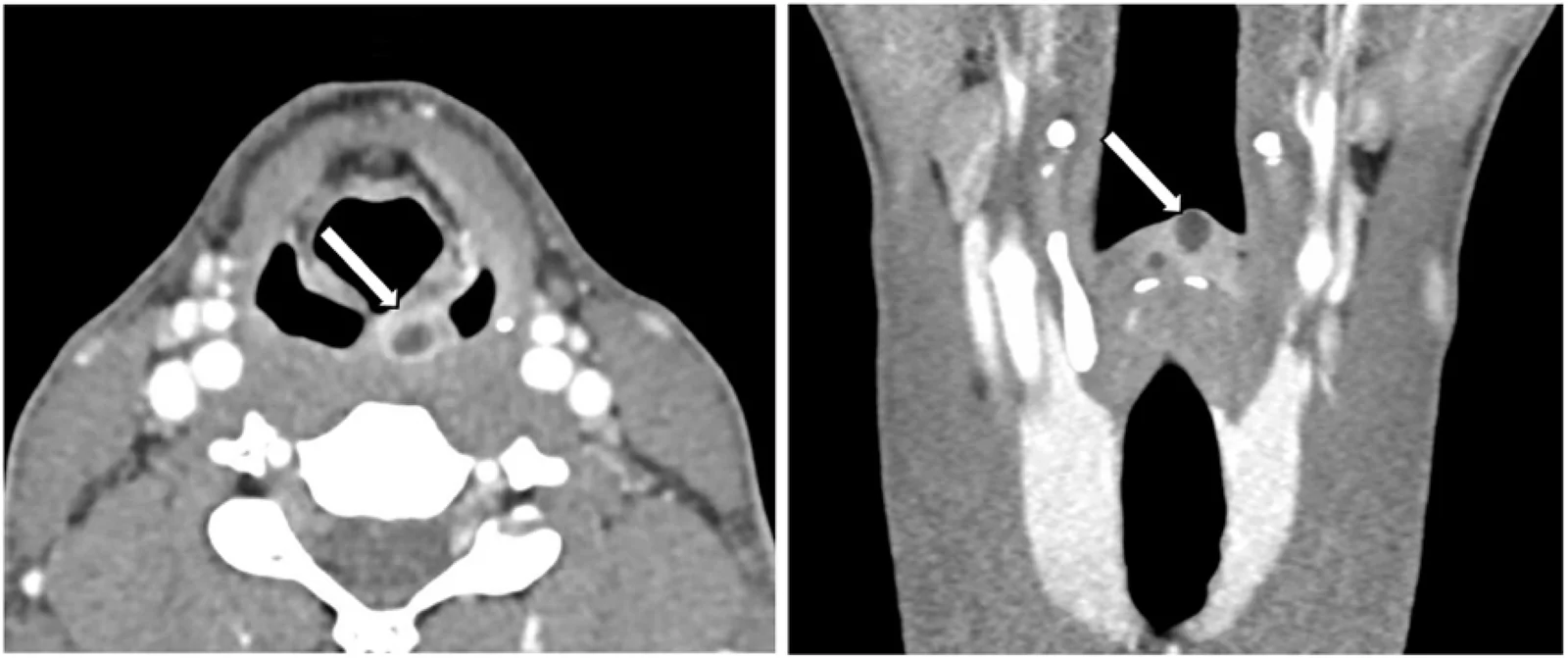
Axial and coronal post-contrast CT images at the time of second hospitalization show reduction of the aryepiglottic fold thickening, more evident on the right, with persistent enhancement on the left. A better-defined cystic lesion with peripheral enhancement is now seen along the inferomedial aspect of the left aryepiglottic fold (arrows).

The patient's sore throat persisted in the following days, along with dehydration. She was hospitalized for three days with intravenous amoxicillin associated with clavulanate and, after discharge, with oral amoxicillin associated with clavulanate. After three days, the patient's sore throat worsened, preventing oral intake of liquids and food, and a second hospitalization was decided upon. Ceftriaxone, Clindamycin, and corticosteroids were administered. Follow-up CT demonstrated a reduction in the thickening of the aryepiglottic folds, more pronounced on the right side. On the left side, however, persistent increased enhancement remained, consistent with ongoing inflammatory or infectious changes. A small cystic-appearing lesion with peripheral contrast enhancement became more conspicuous along the inferomedial aspect of the left aryepiglottic fold (Fig. 2).

**Fig. 3:**
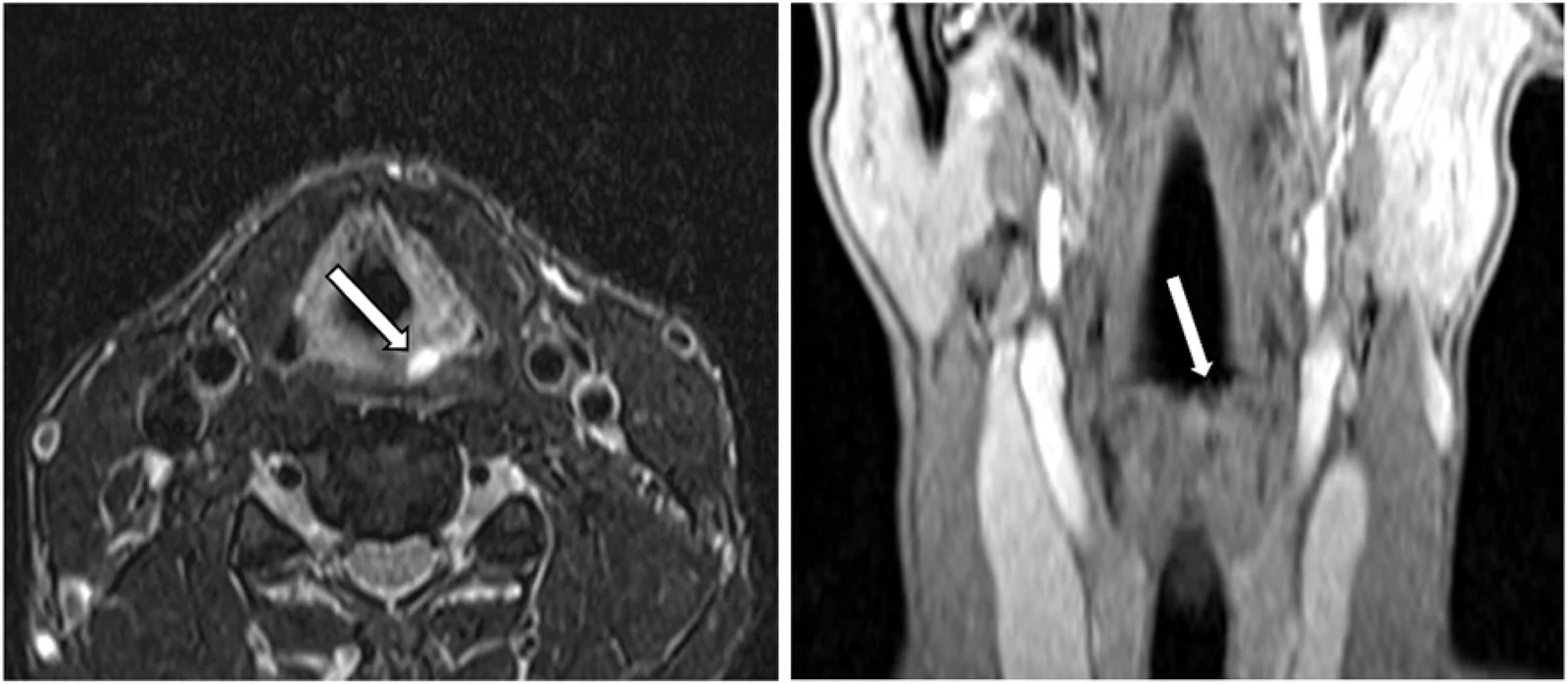
Axial T2-weighted fat-suppressed and coronal post-contrast T1-weighted MR images performed during the second hospitalization demonstrate marked reduction of the inflammatory changes in the aryepiglottic folds, with persistence of a small cystic lesion along the left aryepiglottic fold, without associated inflammatory signs (arrows).

The patient’s condition improved, and three days later a neck MRI demonstrated further reduction in the thickening and edema of the aryepiglottic folds, now appearing only mildly prominent. A thin-walled cystic lesion was still observed in the posteroinferior portion of the left aryepiglottic fold, measuring approximately 0.7 × 0.4 cm, without peripheral enhancement or diffusion restriction, and no associated inflammatory changes (Fig. 3). The patient was discharged one day later with clindamycin and cefuroxime and referred for my evaluation.

**Fig. 4:**
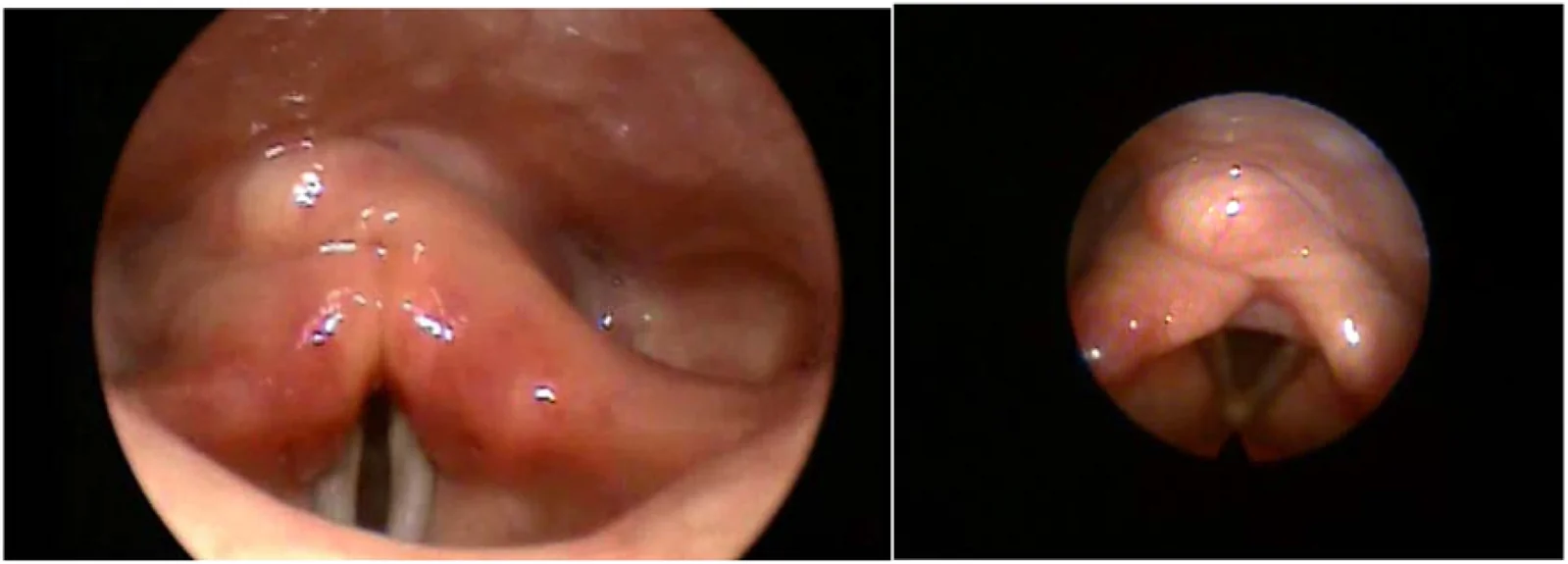
Telelaryngoscopy and nasofibrolaryngoscopy performed during the first assessment on the day of discharge. Laryngoscopy revealed edema of the retro-cricoid region and a cystic-like lesion in the right retro-cricoid region but with insertion on the left side.

**Fig. 5:**
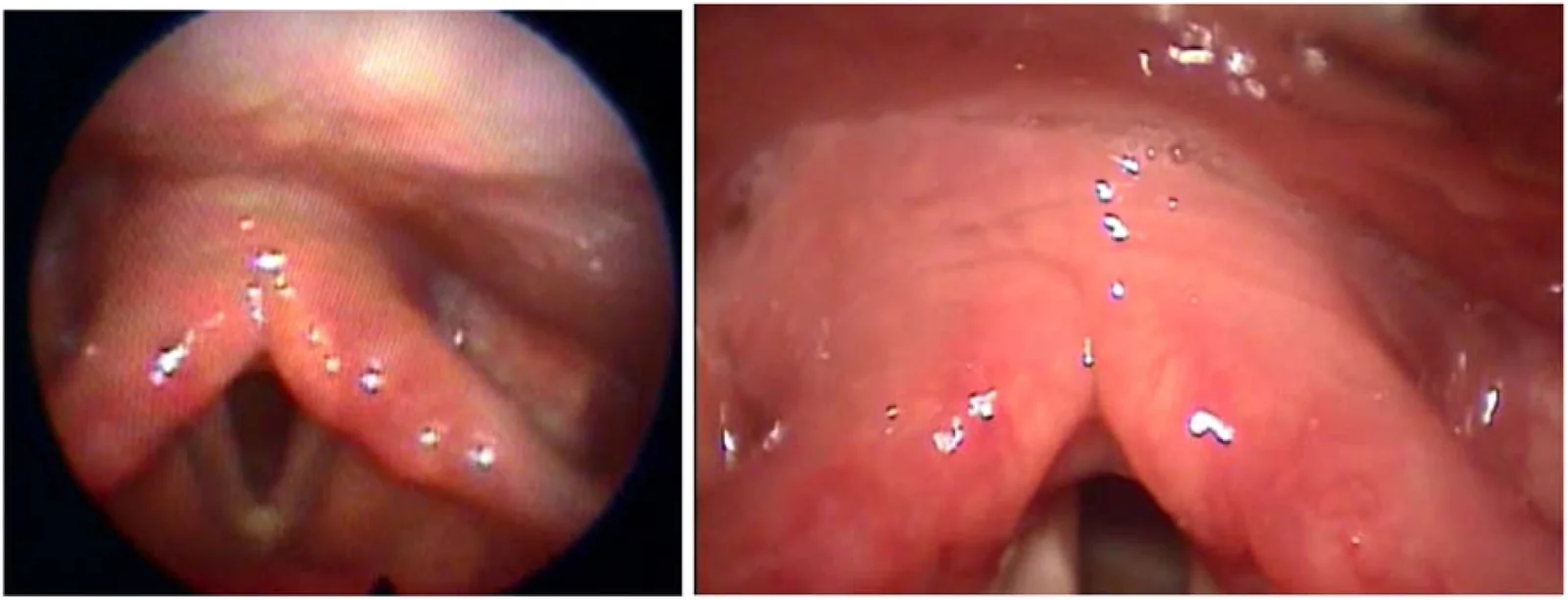
Nasofibrolaryngoscopy after 10-days and telelaryngoscopy after 1-month. No changes were observed in the posterior region of the larynx.

In my evaluation, the patient presented mild throat discomfort, and laryngoscopy revealed edema of the retro-cricoid region and a cystic-like lesion in the right retro-cricoid region but with insertion on the left side (Fig. 4). The patient was instructed to return after 10-days, one month, and two months, during which the patient was asymptomatic, and no changes were observed in the posterior region of the larynx (Fig. 5). Between the first and second month, the patient was submitted to tonsillectomy because of several episodes of tonsillitis.

## Discussion

This case demonstrates the importance of a more comprehensive evaluation in patients with persistent sore throats even after improvement observed the oropharyngeal physical examination. Flexible endoscopy allows evaluation from the nasal cavity to the hypopharynx and larynx, allowing assessment of areas that may also present an acute inflammatory process resulting in pain.

Some reports cited respiratory distress in patients with aryepiglottic cyst.^2^^,^^3^ Due to the small size of the current case, the only symptom was pain. One case report described an aryepiglottic cyst that developed hours after tracheal intubation.^3^ Our patient was submitted to tracheal intubation and did not present any laryngeal symptoms after surgery (tonsillectomy).

According to the classification proposed by Heyes and Lott, the cyst described in the present study was categorized as a supraglottic submucosal cyst, NOS (Not Otherwise Specified), defined as “a large supraglottic submucosal cystic lesion of unknown etiology or true anatomic location”.^1^

Aryepiglottic cyst is not a common finding and in a recently proposed diagnostic criteria it could be classified as a supraglottic submucosal cyst not otherwise specified, although this classification considers a large supraglottic submucosal cystic.^1^

## Conclusion

Aryepiglottic cyst is not common finding and should be suspected in patients with persistent sore throat even with normal oropharyngeal physical examination.

## ORCID ID

Gustavo Polacow Korn: 0000-0003-3718-204X

Fernando Pochini Sobrinho: 0009-0002-9905-9979

Hugo Luis de Vasconcelos Chambi Tames: 0000-0002-1323-8355

## Funding

None.

Data availability statement

The authors declare that all data are available in repository.

## Declaration of competing interest

The authors declare no conflicts of interest.
